# A noninvasive and highly sensitive approach for the assessment of coronary collateral circulation by 192-slice third-generation dual-source computed tomography: Retraction

**DOI:** 10.1097/MD.0000000000048923

**Published:** 2026-05-08

**Authors:** Kebin Chen, Xiaoge Zhang, Daling Li, Honglei Chen, Zhixu Zhang, Lei Chen

The article, “A noninvasive and highly sensitive approach for the assessment of coronary collateral circulation by 192-slice third-generation dual-source computed tomography”^1^, which appears in Volume 98, Issue 38 of *Medicine*, is being retracted. After publication, a concerned reader alerted the Editorial Office of an investigation conducted by the National Natural Science Foundation of China. As part of our investigation, we contacted their division of research ethics, and the head of the division confirmed that the authors were found with misused funding numbers, paper trading and submission by third party, but they could not share the investigation details with us according to regulations. The authors were contacted to respond to the investigation and agreed to the retraction in light of the investigation as well as over concerns over the reliability of the original data. As a result, the Publisher no longer has confidence in the integrity of the content.
